# The Genetic Variants of NOTCH3 (6746T>C) and PSMA6 (-8C>G) as Possible Risk Factors of Psoriasis Development

**DOI:** 10.3390/life11090887

**Published:** 2021-08-28

**Authors:** Joanna Bartosińska, Szymon Zmorzyński, Beata Sarecka-Hujar, Dorota Raczkiewicz, Magdalena Wojcierowska-Litwin, Iwona Korszeń-Pilecka, Anna Michalak-Stoma, Małgorzata Kowal, Jarosław Bartosiński, Agata Filip, Dorota Krasowska, Grażyna Chodorowska

**Affiliations:** 1 Department of Cosmetology and Aesthetic Medicine, Medical University of Lublin, 20-093 Lublin, Poland; 2 Department of Cancer Genetics with Cytogenetic Laboratory, Medical University of Lublin, 20-080 Lublin, Poland; s.zmorzynski@gmail.com (S.Z.); magdalena.wojcierowska-litwin@umlub.pl (M.W.-L.); iwona.korszen-pilecka@umlub.pl (I.K.-P.); agata.filip@umlub.pl (A.F.); 3 Department of Basic Biomedical Science, Faculty of Pharmaceutical Sciences in Sosnowiec, Medical University of Silesia, 41-200 Sosnowiec, Poland; bsarecka-hujar@sum.edu.pl; 4 Department of Medical Statistics, School of Public Health, Center of Postgraduate Medical Education, 01-826 Warsaw, Poland; dorota.bartosinska@gmail.com; 5 Department of Dermatology, Venereology and Pediatric Dermatology, Medical University of Lublin, 20-081 Lublin, Poland; annamichalak@wp.pl (A.M.-S.); kowalma71@o2.pl (M.K.); dor.krasowska@gmail.com (D.K.); grazynach90@gmail.com (G.C.); 6 Department of Anaesthesiology and Intensive Therapy, Independent Public Clinical Hospital No. 4 in Lublin, 20-097 Lublin, Poland; jeremybartosinski@gmail.com

**Keywords:** psoriasis, genetics of psoriasis, NOTCH3 polymorphism, PSMA6 polymorphism

## Abstract

Advances in genotypic technologies enable identification of possible associations between genetic variants of certain genes and increased risk of developing plaque psoriasis or psoriatic arthritis. The aim of the study was to analyze the *NOTCH3* (6746T>C) (rs1044009) and *PSMA6* (-8C>G) (rs1048990) polymorphisms and their role in genetic susceptibility to psoriasis. The study included 158 psoriatic patients and 100 healthy controls. The frequencies of the *NOTCH3* genotypes differed between the psoriatic patients and healthy controls (*p* = 0.050). No differences were found in the distribution of *PSMA6* genotypes and alleles between the psoriatic patients and healthy controls. The studied psoriatic patients presented a higher frequency of the CC genotype of *PSMA6* compared to the healthy controls (8.8% vs. 2%, respectively). Psoriatic arthritis was more frequent among patients with the CC genotype of *PSMA6* (*p* = 0.059). CC homozygosity of *NOTCH3* was more commonly observed in the studied psoriatic patients than in the healthy controls (OR = 4.76, *p* **= 0.032**). The obtained data suggest that genetic variants of *NOTCH3* (6746T>C) and *PSMA6* (-8C>G) genes may play significant roles in psoriatic patients. Further studies are necessary to unequivocally determine their role as genetic risk factors of psoriasis development.

## 1. Introduction

Genotyping technologies have made it possible to identify numerous single nucleotide polymorphisms (SNPs) which are helpful in detecting genetic markers of multiple pathologies, inter alia, psoriasis [1,2]. The genetic background of psoriasis accounts for the uncontrolled proliferation and abnormal differentiation of keratinocytes resulting from disturbances in the innate and adaptive cutaneous immune responses. The interplay between keratinocytes and immune cells, including Th1, Th17, regulatory T cells (Tregs), and neutrophils, expressed by activation of the TNFα–IL-23–Th17 proinflammatory axis, is known to cause changes in both the epidermis and dermis, i.e., epidermal hyperplasia, dermal inflammatory infiltration as well as elongation and increased permeability of blood vessels [3,4,5].

The NOTCH signaling pathway, a highly conserved cell signaling system involving interaction between the NOTCH receptors and their ligands, has been shown to play an important role in the regulation of cell proliferation, differentiation, migration, and apoptosis. Some authors suggest that the NOTCH signaling pathway is also involved in the development of various skin diseases, including psoriasis, atopic dermatitis, hidradenitis suppurativa, Dowling Degos disease, and Adams–Oliver syndrome [6]. The NOTCH proteins form a family of four single-pass type 1 transmembrane receptors encoded by the NOTCH 1, 2, 3, and 4 genes. After their activation by transmembrane ligands, such as Jagged-1/-2 and Delta-like 1, 3, and 4, these NOTCH proteins are ready to transduce cellular signals. However, the overall outcome of the NOTCH signaling is highly dependent on the interaction of cellular mechanisms involved in various pathogeneses, especially in inflammation or malignancy [6,7].

In psoriatic patients, Rooney et al. [8] observed hyperproliferation of the epidermal cells resulting from upregulation of Jagged-1 expression and activation of the NOTCH signaling pathway. On the other hand, according to Ota et al. [9], a lack of NOTCH signaling causes abnormal differentiation of keratinocytes observed in psoriasis. Nickoloff et al. [10] found that the Notch-1 receptor and its peptide Delta-like 1 ligand were able to regulate early differentiation of keratinocytes. Apart from its effect on the epidermal cells, NOTCH is also important in the development of the T cells and their function [11]. Some researchers observed that the NOTCH ligands are involved in polarizing the T cell response towards producing Th1 and Th17 cells upon induction of the Delta-like ligands on the dendritic cells (DCs). However, when NOTCH interacts with the Jagged ligands, it activates production of Th2 cells [11,12]. This suggests that the NOTCH signals can alter the balance of the CD4 T cell differentiation into the Th1 or Th2 lineage. Furthermore, Pan M et al. [13] suggested that in psoriasis, the activation of the Notch signaling pathway may be regulated by miR-125b, a molecule engaged in the control of cell proliferation by downregulation of the Jagged-1 ligand expression. NOTCH activation is also involved in different stages of blood vessel development, including vascularization and angiogenesis [14,15,16]. Therefore, the NOTCH signaling pathway is supposed to play a role in psoriasis by modulation of keratinocyte proliferation, immune processes, and angiogenesis. While the role of NOTCH1 in the skin is fairly well explained, there is still little information about a possible role of NOTCH3 in the pathogenesis of skin diseases. NOTCH3, whose transcription can be directly activated by the intracellular domain of NOTCH1, is another regulator of keratinocyte differentiation controlling involucrin expression at the late phase of the process [17,18]. Lopez-Lopez et al. [19] revealed the role of NOTCH3 expression and signaling in proinflammatory macrophage development and in the activation of the proinflammatory nuclear factor ĸB (NF-ĸB) signaling pathway. The NOTCH3 receptor also participates in the regulation of T cell differentiation and may be engaged in the autoinflammatory processes in psoriasis [20]. 

Similarly to the Notch pathway, the proteasome system, a large multiple subunit enzyme complex, also controls apoptosis, proliferation, differentiation, and inflammation. PSMA6 is coded by the gene located on the chromosome 14q13.2 and is a component of the 20S proteasome complex, which is the main pathway for degradation of oxidatively damaged proteins [21]. There is a single PSMA6 (-8C>G) (rs1048990) nucleotide polymorphism in the exon 1 which has been found to be associated with diabetes, myocardial infarction, and coronary artery disease also known as “oxidative stress conditions” [22]. Therefore, since psoriasis is a systemic disease, it may be surmised that the PSMA6 polymorphism plays a role in the genetic susceptibility to psoriasis. The presence of the G allele is associated with the PSMA6 transcriptional augmentation. An increased PSMA6 activity aggravates inflammation through activation of the NF-ĸB signaling pathway [23]. In psoriasis, activation of the NF-κB signal induces expression of keratins 6 and 16 in keratinocytes, which leads to acanthosis and shortened turnover time in the epidermis [24]. Both expression and activity of the 20S proteasome are also increased in the psoriatic skin cells [5]. Stuart et al.’s meta-analysis of two genome-wide association studies (GWAS) revealed three genomic regions associated with psoriasis, one contained NOS, another one contained FBXL19, and the third one contained PSMA6 and NFKBIA. All of them were associated with both plaque psoriasis and psoriatic arthritis. Among them only PSMA6, an encoding proteosomal subunit involved in MHC Class I antigen processing, was overexpressed in psoriatic lesions [25]. Moreover, in the blood of psoriatic arthritis patients, Colmegna et al. [26] observed elevated levels of anti-proteasome antibodies, which, according to them, might reduce the activity of proteasomes. Reduction in the 20S proteasome activity in the blood cells of psoriatic patients observed by Karabowicz et al. [5] confirmed the findings of Colmegna et al. [26]. 

In the light of the literature search we performed, it appears that NOTCH3 and PSMA6 gene polymorphisms require further investigation. Therefore, our study made an attempt to find a possible association between the NOTCH3 as well as PSMA6 polymorphisms and genetic susceptibility to psoriasis. Moreover, since in our literature search we did not come across information about a possible genetic interplay between the SNPs of NOTCH3 (6746T>C) and PSMA6 (-8C>G) in psoriasis, the aim of this study was to analyze the NOTCH3 (6746T>C) and PSMA6 (-8C>G) polymorphisms and the role they play in genetic susceptibility to psoriasis in Polish psoriatic patients.

## 2. Materials and Methods

### 2.1. Study Groups

The study consisted of 158 psoriatic patients and 100 healthy controls. Patients were recruited in the Chair and Department of Dermatology, Venereology and Pediatric Dermatology, the Medical University of Lublin, Poland, from May 2018 to May 2020. The inclusion criteria for patients were as follows: (a) age ≥18 years; (b) confirmed psoriasis; (c) unrelated individuals; (d) Caucasian race. Healthy subjects were recruited from a group of blood donors in the Regional Blood Donation and Blood Treatment Center in Kielce, Poland, and they had to fulfill the following inclusion criteria: (a) age ≥18 years; (b) Caucasian race. Blood donors were excluded in the cases of: (a) HIV infection, syphilis, tuberculosis, hepatitis B, or hepatitis C; (b) condition that requires active medical intervention or monitoring to avert serious danger to the participant’s health or wellbeing; (c) race other than Caucasian.

The study was approved by the local ethics committee of the Medical University of Lublin (KE-0254/35/2018). All recruited patients and healthy controls gave written informed consent to participate in the study. 

### 2.2. DNA Isolation and Genotyping of NOTCH3 6746T>C and PSMA6 -8C>G Polymorphisms

DNA isolation from peripheral blood was performed using a commercial kit (Qiagen, Hilden, Germany) according to the manufacturer’s procedure. The concentration and quality of DNA were checked using a NanoDrop device (Thermo Fisher Scientific, Waltham, MA, USA). The *NOTCH*3 polymorphism was assessed by the PCR-restriction fragment length polymorphism (RFLP) method. Each PCR mix (25µL) contained 150 ng genomic DNA, and PCR buffer (Clontech Laboratories, Mountain View, CA, USA), dNTPs mix (0.25 mM), HD polymerase (Clontech Laboratories, Mountain View, CA, USA) and primers (10 µM of each). The mix was heated to 94 °C for 5 min and underwent 35 cycles of amplification: denaturation at 98 °C for 10 s, annealing at 64 °C for 10 s, elongation at 72 °C for 20 s. The final elongation took 5 min at 72 °C. The PCR reaction was performed using an Applied Biosystems 9700 Thermal Cycler. The following primers were used in PCR reaction:

-forward 5′-CTT ACC TGG CAG TCC CAG G-3′

-reverse 5′-AGT GGC AGT GGC TGG GCT AG-3′

**Figure 1:**
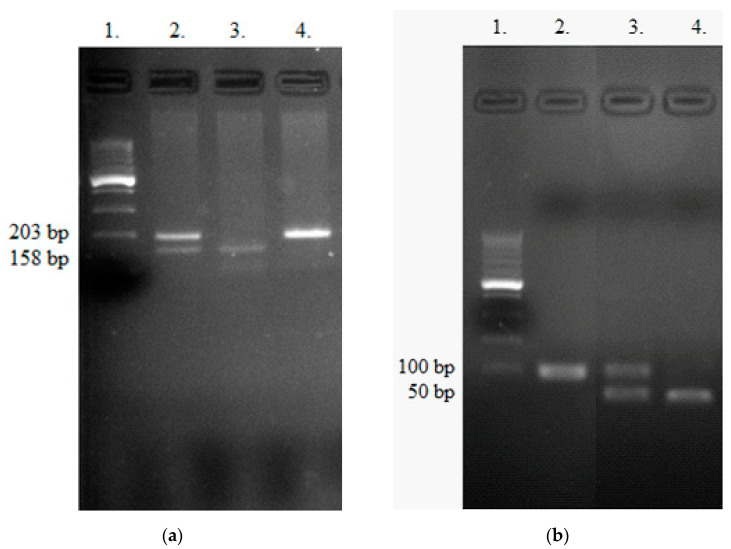
Detection of 6746T>C *NOTCH3* (**a**) and -8C>G *PSMA6* (**b**) polymorphisms. (**a**) Lane 1—Ladder (100bp); Lane 4 contains TT genotype; Lane 2 contains TC heterozygote; Lane 3 contains CC genotype. (**b**) Lane 1—Ladder (100bp); Lane 2 shows CC genotype; Lane 3—CG heterozygote, Lane 4—GG homozygote.

The PCR products were digested with *MwoI* (HpyF10VI) restriction enzyme (Thermo Fisher Scientific, Waltham, MA, USA) for 16 h at 37 °C. The RFLP products were analyzed on 3% agarose gel, stained with SimplySafe (Eurx, Gdansk, Poland) and visualized in G:Box (Syngene, Great Britain). T or C alleles were identified by the presence of 203 bp (TT genotype) or 158 bp (CC genotype) fragments, respectively. Heterozygous TC genotype showed the presence of two bands—158 bp and 203 bp (Figure 1a). An independent PCR analysis was carried out for each sample.

For an analysis of the *PSMA6* polymorphism, the PCR–RFLP method was applied according to the validated protocol of Bachmann et al. 2010 [27]. *PSMA6* gene fragment length of 100 bp was amplified in PCR reaction using the following primers: 

-forward 5′-CTG GTG CGG GAG CTA CGG G-3′

-reverse 5′-AAT GGT AAT GTG GCG GTC AAA AC-3′

Each PCR mixture (25 µL) contained 100 ng genomic DNA and PCR buffer (Clontech), dNTPs mixture (0.25 mM), HD polymerase (Clontech) and primers (10 µM of each). The touchdown PCR method was used. The mixture was heated at 95 °C for 5 min and underwent 14 cycles of amplification: denaturation at 95 °C for 30 s, annealing at 64.5 °C for 20 s (−0.5 °C/per cycle), elongation at 72 °C for 20 s. After 14 cycles, the mixture underwent 20 cycles with the constant temperature of 57.5 °C. The denaturation and elongation temperatures and times were the same as above. The final elongation took 5 min at 72 °C. The PCR reaction was performed in an Applied Biosystems 9700 Thermal Cycler. The PCR product was digested with *RsaI* (Thermo Fisher Scientific, Waltham, MA, USA) for 16 h at 37 °C producing two fragments of 50 bp or one fragment 100 bp for the presence of G or C allele, respectively. The RFLP products were analyzed on 3% agarose gel and stained with SimplySafe (Eurx, Gdansk, Poland) and visualized in G:Box (Syngene, Great Britain) (Figure 1b). An independent PCR analysis was carried out for each sample.

### 2.3. Statistical Methods

The data were statistically analyzed using Statistica 13.1 software (STATSOFT, Tulsa, OK, USA). Mean values (M) and standard deviations (SD) were estimated for continuous variables, while absolute numbers (*n*) and relative numbers (%) were estimated for categorical variables. The allele frequencies were assessed on the basis of the genotype distribution. The Hardy–Weinberg equilibrium (HWE) was evaluated in the healthy controls by an *χ*^2^ test. Fisher’s exact test was used to compare genetic models between the patients and healthy controls. If a significant difference was detected, the odds ratio (OR) was estimated. In correlation analyses between genetic models and psoriasis features, the Mann–Whitney’s U test or Fisher’s exact test were used because some expected counts in cross tables were smaller than 5. The significance level was set at *p* ≤ 0.05 in all statistical tests.

## 3. Results

### 3.1. Characteristics of the Patients

In the study, 158 psoriatic patients and 100 healthy controls were analyzed. Table 1 shows the characteristics of the studied psoriatic patients and healthy controls. The mean age of the patients at the onset of psoriasis was 23.3 ± 12.3 years. In 86.71% of the patients, psoriasis developed at or under the age of 40 years (Type 1 psoriasis), while in the remaining 13.29% patients it started when they were over 40 (Type 2 psoriasis). The mean psoriasis duration was 23.3 ± 12.5 years. A total of 35.44% of patients suffered from psoriatic arthritis. In 74.68% of the patients, psoriasis was moderate or severe. Almost half of the studied patients (49.63%) had a positive family history of psoriasis.

### 3.2. Frequencies of Genotypes and Alleles of NOTCH3 and PMSA6 Polymorphisms

**Figure 2:**
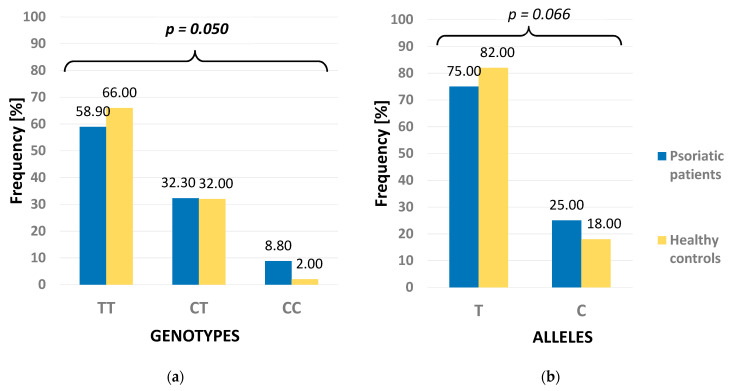
Frequency of genotypes (**a**) and alleles (**b**) of NOTCH3 polymorphism in psoriatic patients and healthy controls. *p* for Fisher’s exact test.

**Figure 3:**
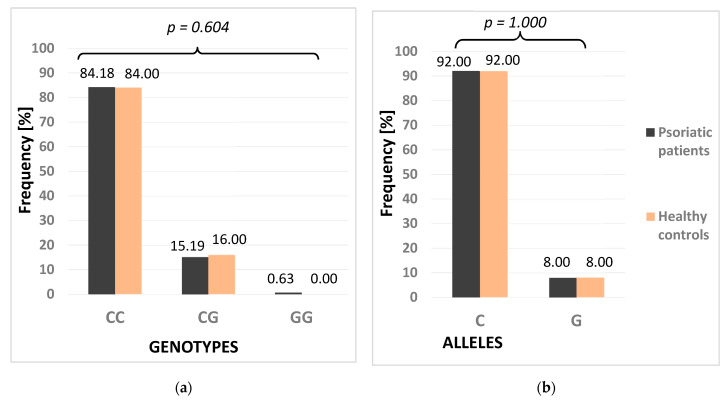
Frequency of genotypes (**a**) and alleles (**b**) of PMSA6 polymorphism in psoriatic patients and healthy controls. *p* for Fisher’s exact test.

Genotyping analyses were successful in all the studied subjects. Distribution of the NOTCH3 genotypes in the control group was in agreement with the Hardy–Weinberg Equilibrium (HWE) model (χ^2^ = 0.706, *p* = 0.401). Similarly, distribution of the PSMA6 genotypes was also in agreement with HWE (χ^2^ = 0.756, *p* = 0.385). The frequencies of the NOTCH3 genotypes differed between the patients and healthy controls (*p* = 0.050) (Figure 2). However, no differences were demonstrated in the distribution of PSMA6 genotypes and alleles between the psoriatic patients and healthy controls (Figure 3). The studied psoriatic patients presented a higher frequency of the CC genotype compared to the healthy controls (8.8% vs. 2%, respectively). No statistical difference was found when the distribution of alleles was analyzed.

### 3.3. NOTCH3 and PSMA6 Polymorphisms and Psoriasis in the Analysis of Genetic Models 

The relationship between the NOTCH3 polymorphism and psoriasis was analyzed using five genetic models: dominant (TC + CC vs. TT), recessive (CC vs. TT + TC), additive (CC vs. TT), heterozygote (TC vs. TT), and allelic (C vs. T). In the recessive model, a significant difference was demonstrated (*p* = 0.001). The CC homozygotes were more frequent in the patient group than in healthy controls (8.86% vs. 2%, respectively). The individuals being CC homozygotes had a five-fold higher risk of the disease (OR = 4.76 95%CI 1.06–21.43, *p* = 0.032). Similarly, a significant difference was observed in the case of the additive model of NOTCH3 polymorphism (OR = 4.968 95%CI 1.09–22.60, *p* = 0.030). In the allelic model, the C allele was found to be more prevalent in the psoriatic patients compared to healthy controls (25% vs. 18%, respectively), however, the result was close to a statistical significance (*p* = 0.066).

The same genetic models were also used for analyses of the PSMA6 polymorphism in the psoriatic patients and healthy controls: dominant (CG + GG vs. CC), recessive (GG vs. CG + CC), additive (GG vs. CC), heterozygote (CG vs. CC), and allelic (G vs. C). No association between the PSMA6 polymorphism and psoriasis was observed in any of the used genetic models. Table 2 demonstrates the results of analyses of genetic models for both investigated polymorphisms.

### 3.4. Joint Analysis of NOTCH3 and PSMA6 Polymorphisms 

Comparison of the impact of NOTCH3 and PSMA6 polymorphisms on the risk of psoriasis is demonstrated in Table 3. The combinations of genotypes of both polymorphisms did not differ significantly between the psoriatic patients and healthy controls. 

### 3.5. Correlations between Analyzed Polymorphisms and Psoriasis Clinical Features

The correlation of PSMA6 G allele carriers (GG + CG) and CC homozygotes with psoriasis clinical features revealed that psoriatic arthritis was more frequent among patients with the CC genotype (*p* = 0.059). The other psoriasis clinical features did not correlate with the PSMA6 polymorphism. Similarly, no statistical correlations were found between the NOTCH3 polymorphism and psoriasis parameters (Table 4). 

## 4. Discussion

It goes without saying that genetic, immune, and environmental factors conspire with one another to trigger psoriasis. Our study aimed at investigating the genetic aspect of psoriasis etiology and it focused especially on the NOTCH3 and PSMA6 polymorphisms. We made an attempt to detect possible links between the NOTCH3 (6746T>C) and PSMA6 (-8C>G) polymorphisms and psoriasis in five genetic models: dominant, recessive, additive, heterozygote, and allelic. 

The results of our study showed a higher frequency of the NOTCH3 CC genotype in the psoriatic patients in comparison to healthy controls (*p* = 0.050), whereas the other investigated genotypes and alleles were similar in both studied groups. Compared with the healthy controls, the recessive (CC vs. TT + TC) and additive (CC vs. TT) models of the NOTCH3 polymorphisms were significantly different in the psoriatic patients. The CC homozygotes were more frequent in the studied patients than in healthy controls and the individuals with CC homozygotes had an approximately five-fold higher risk of developing psoriasis. Moreover, the allelic model was more often observed in the studied psoriatic patients, and this finding turned out to be close to statistical significance (*p* = 0.066). In light of the study of Ota et al. [9], who investigated Notch1, 2, and 3 on mRNA and protein levels and observed their decreased expressions in the psoriatic epidermis compared with normal epidermis, our study results may be supportive of a role of the NOTCH3 (6746T>C) polymorphism in the pathogenesis of psoriasis.

Investigation of the other studied polymorphism, PSMA6 (-8C>G), brought to light no links between the PSMA6 genotypes and alleles and psoriasis. However, in our patients with psoriatic arthritis, the PSMA6 CC genotype was more frequently observed than in the carriers of G allele (38% vs. 20%). The difference was found to be close to statistical significance (*p* = 0.059), while the other studied clinical features did not correlate with the PSMA6 polymorphism. This observation is quite surprising since the G allele was demonstrated to enhance the transcription of PSMA6, also in the HEV cell lines. The authors suggested that some nuclear factor(s) may bind to this region and thus regulate transcription of PSMA6. In turn, an altered expression of PSMA6 gene may increase inflammation through activation of the NF-kB protein [23]. Similar to our results, the PSMA6 CC genotype was previously demonstrated to be more common in the patients with the endstage of renal disease than in controls (80% vs. 58%, respectively), which suggested a protective role of the G allele [28]. In our study, the PSMA6 G allele carrier frequency was similar in the psoriatic patients and healthy controls. Only one psoriatic patient was a GG homozygote, which may be a result of a limited number of study subjects. Our study results appear to support the view that a reduced activity of proteasome may be somehow associated with the PSMA6 polymorphism.

We made an interesting observation that in the individuals who were NOTCH3 CC homozygotes, the risk of psoriasis was almost a five-fold higher than in the individuals with other combinations of genotypes (OR = 4.76). The NOTCH3 polymorphism results in an amino acid dimorphism (Val/Ala) at residue 2223 of the intracellular domain. Since the intracellular domain of NOTCH3 is thought to be involved in signal transduction, this polymorphism has been suggested to be directly associated with the NOTCH3 function, i.e., keratinocyte and T cell differentiations [15,29]. 

To the best of our knowledge, this study has made the first attempt to shed more light on the relationship between the PSMA6 polymorphism and psoriasis. Nevertheless, the study has a few limitations. Firstly, in order to establish the role of PSMA6 polymorphism in the development of psoriasis, further experimental studies are necessary. Secondly, our study was performed on a limited number of psoriatic patients all of whom were Caucasian. 

## Figures and Tables

**Table 1:** The characteristics of psoriatic patients and healthy controls.

Variable, Parameter	Unit or Category	Psoriatic Patients (*n* = 158)	Healthy Controls (*n* = 100)	*p*
Age, Min–Max, M ± SD	years	20–84, 46.5 ± 14.1	18–61, 34.3 ± 11.4	<0.001
Gender, *n* (%)	men	112 (70.89)	50 (50.00)	0.001
	women	46 (29.11)	50 (50.00)	
Age of psoriasis onset, Min–Max, M ± SD	years	1–63, 23.3 ± 12.3		
Psoriasis subtype, *n* (%)	at/under 40 years of age	137 (86.71)		
	over 40 years of age	21 (13.29)		
Psoriasis duration, Min–Max, M ± SD	years	2–52, 23.3 ± 12.5		
Psoriatic arthritis, *n* (%)	yes	56 (35.44)		
Severity of psoriasis, *n* (%)	mild	40 (25.32)		
	moderate or severe	118 (74.68)		
Positive family history, *n* (%) (*n* = 135)	yes	67 (49.63)		

M—mean, SD—standard deviation, *p* for age—Mann–Whitney’s U test, *p* for gender—chi-square test.

**Table 2:** The analyses of the relation between NOTCH3 and PSMA6 polymorphisms and psoriasis using genetic models.

Polymorphism	Genetic Model	Genotypes/Alleles	Psoriatic Patients (*n* = 158) *n* (%)	Healthy Controls (*n* = 100) *n* (%)	*p*
*NOTCH3*	Dominant	TC + CC	65 (41.14)	34 (34.00)	0.293
		TT	93 (58.86)	66 (66.00)	
	Recessive	CC	14 (8.86)	2 (2.00)	0.032
		TT + TC	144 (91.14)	98 (98.00)	
	Additive	CC	14 (13.08)	2 (2.94)	0.030
		TT	93 (86.92)	66 (97.06)	
	Heterozygote	TC	51 (35.42)	32 (32.65)	0.681
		TT	93 (64.58)	66 (66.35)	
	Allelic	C	79 (25.00)	36 (18.00)	0.066
		T	237 (75.00)	164 (82.00)	
*PSMA6*	Dominant	CG + GG	25 (15.82)	16 (16.00)	1.000
		CC	133 (84.18)	84 (84.00)	
	Recessive	GG	1 (0.63)	0 (0.00)	1.000
		CC + CG	157 (99.37)	100 (100.00)	
	Additive	GG	1 (0.75)	0 (0.00)	1.000
		CC	133 (99.25)	84 (84.00)	
	Heterozygote	CG	24 (15.29)	16 (16.00)	1.000
		CC	133 (84.71)	84 (84.00)	
	Allelic	G	25 (8.00)	16 (8.00)	1.000
		C	291 (92.00)	184 (92.00)	

*p* for Fisher’s exact test.

**Table 3:** Comparison of the impact of NOTCH3 and PSMA6 polymorphisms on the risk of disease. (*p = 0.236* for Fisher’s exact test).

NOTCH3 Genotypes	PSMA6 Genotypes	Psoriatic Patients (*n* = 158) *n* (%)	Healthy Controls (*n* = 100) *n* (%)
TT	CC	79 (50.00)	58 (58.00)
TC	CC	40 (25.32)	24 (24.00)
CC	CC	14 (8.86)	2 (2.00)
TT	CG	14 (8.86)	8 (8.00)
TC	CG	10 (6.33)	8 (8.00)
CC	CG	0 (0.00)	0 (0.00)
TT	GG	0 (0.00)	0 (0.00)
TC	GG	1 (0.63)	0 (0.00)
CC	GG	0 (0.00)	0 (0.00)

**Table 4:** Correlations of *NOTCH3* and *PSMA6* polymorphisms with psoriasis characteristics.

Variable, Category, Parameter	*NOTCH3* Polymorphism			*PSMA6* Polymorphism		
	C Allele Carriers (CC + TC) (*n* = 65)	Wild-Type Homozygotes (TT) (*n* = 93)	*p*	G Allele Carriers (GG + CG) (*n* = 25)	Wild-Type Homozygotes (CC) (*n* = 133)	*p*
Gender, male, *n* (%)	46 (70.77)	66 (70.97)	0.558	18 (72.00)	94 (70.68)	0.551
Age of onset, M ± SD	22.9 ± 11.2	23.6 ± 13.1	0.727	23.7 ± 12.8	23.2 ± 12.3	0.855
Psoriasis subtype, over 40 years of age, *n* (%)	6 (9.23)	15 (16.13)	0.154	3 (12.00)	18 (13.53)	0.567
Psoriasis arthritis, *n* (%)	25 (39.68)	31 (33.33)	0.310	5 (20.00)	51 (38.35)	0.059
Severity, moderate or severe, *n* (%)	47 (72.31)	71 (76.34)	0.347	20 (80.00)	98 (73.68)	0.348
Positive family history, *n* (%) *	27 (49.09)	40 (50.00)	0.528	13 (54.17)	54 (48.65)	0.396

* Note: missing data in every column: 1, 22, 10, and 13, respectively. *p* for Fisher’s exact test or Mann–Whitney’s U test.

## Data Availability

The data presented in this study are available on request from the corresponding author.
